# Editorial and Miscellaneous Correspondence

**Published:** 1872-02

**Authors:** 


### EDITORIAL & MISCELLANEOUS CORRESPONDENC.

We yield our editorial space in this number to the members of our editorial staff, and other friends. It will be remembered by our readers that, a few months since we invited our friends, and the friends of "law and order," everywhere, to write brief points on every subject connected with the welfare of our profession. In response to this appeal, we have received a number of paragraphs and letters, which we cull and present to our readers for their consideration and reflection. The great medical pulse of the country beats in unison to the high demands of the times in elevating and purifying our noble profession.

We still open the COMPANION to the Profession. Through its columns the pure and good men can advocate the cause of truth, principle and law. Through it they may exhort their brethren to good deeds and noble efforts for the upbuilding of a common cause. We, therefore, invite our friends—those true to the honor and integrity of medicine—to send us their views. These "waifs" drawn from the pure hearts of the true men of our profession, will accomplish much in the mighty efforts now being made to winnow the chaff from the wheat of the medical profession. We therefore give below the following editorial and miscellaneous correspondence:

"'CHEATING NEVER THRIVES,' is a very true maxim, though one which people are slow to believe. The reason is that we do not contemplate the end from the beginning. We perhaps see the wicked when he is flourishing like a green bay tree, but do not turn again to see that tree when its leaves have all withered, its branches fallen, and decay is eating away the trunk. Many saw Turpin, and it may be, envied his success in villainy, who did not see that career ended at Tyburn, amid the insults of a scoffing multitude. Read history, and you will there learn that the wicked, though they may prosper long, seldom fail to come to wo[illegible]."

"I NOTICE in your editorial for January, that Dr. Roosa states as a reason why Dr. Ruppaner should be expelled from his Society, was, because he "scouted its authority to investigate the case and report upon it to the Society." That is just what the faculty of the Atlanta Medical College did, but added to their "scouting", an insult by charging that the Medical Association of the State was a body assuming to be the Association. For this reason they were required to apologise, which, refusing to do, they were expelled. Yet, notwithstanding this, the Association has been insulted again by a resolution offered, through an ex-member of their body, charging all the previous action of the Association to "personal feeling." At the meeting in Americus, the faculty party not only reiterated the insult in the memorial, but refused to allow five gentlemen to "in vestigate" and settle the trouble.

If the charge made in that resolution was true, it makes those who composed the Association, complained of in it, if influenced by personal feeling, unworthy of professional recognition; and on the other hand, if the charge is not true, then they have been grossly slandered.

If the charges made by Dr. Sayre, against Dr. Ruppaner, were true, he did his duty in arraigning the latter gentlemen before his Society, and he should be sustained by the profession throughout the country. If, however, he slandered Dr. R., then this gentleman has his remedy. Nothing can as effectually sink a man in infamy as to be convicted of willfully slandering the good name and reputation of his professional brethren."

"'I HAD RATHER BE RIGHT THAN PRESIDENT,' was the utterance of a great moral hero, and we would that the sentiment could be adopted by every man who cherishes aspirations for greatness. Too many there are who prefer greatness, let it be attained as it may. For it they are willing to barter away principle, and sacrifice self-respect. They do not apprehend the fact that all true greatness must be the result of goodness. Position, power, fame, are of but little value, if the dictates of morality be disregarded in order to attain them."

"All honorable men, when apologising for an insult offered, not only apologise for the affront, but to make a settlement upon a true basis, will repair all damages which may have resulted from the insult."

Our friend places the above in the true light. We would be surprised to know that any gentleman would act otherwise. Should he do so, we would at once suspect his sincerity. We do not believe it possible for a gentleman to ask to be pardoned for insulting another, and immediately thereafter repeat the insult.

AN unprincipled man will frequently sustain the wrongs of another, because of his prejudice against the one in the right; but a man of Christian integrity will never sustain even a bosom friend in wrong, but will tell him wherein he is wrong, and beg him to get right."

An unprincipled man is the curse of any community. In every trial he will be found wanting, and in every emergency will write himself " unreliable." Such a character is like " he that hath no music in his soul." He should not—and will not, you may depend upon it—"be trusted." Time, the great revealer, will bring to light the "unprincipled man," as certainly as "the fire will burn and the sea will drown." " And then," as Dean Swift says, "and then," where will his " acts and tricks " place him? "Vengance is mine, saith the Lord—I will repay,"—and He will!

" THE pages of your Journal exhibit a truthfulness and candor which I very much admire, and must have a powerful influence in undecieving the minds of those who have been imposed upon by contrary statements in relation to our professional troubles in Georgia. I trust you will continue until all shall have been informed of the facts. Gross deception, we fear, has been attempted. But persist in your efforts, and the truth will be established and made manifest, good men undeceived, bad men convinced of their error, and the honor of our profession maintained."

We *know* that some good men have been imposed upon. Gross deception has been practiced upon men who will, in the great recoil that is surely coming, have as sublime a contempt for the arts of tricksters as our correspondent has at this moment. "There is nothing so secret but it shall be made known," is an adage, the truth of which *time* will fully demonstrate in this matter. *Some* have been *unearthed* already, and time will make the job so complete that our correspondent will be ready to exclaim, " the perverseness of transgressors shall destroy them."

" THERE can be no such thing as neutrality in questions of right. He who withholds his support from the right, does thereby assist the wrong, and makes him an enemy to truth. To be an honest friend to the truth, you must sustain it by word and act. If you do not do so, let your *professions* be as they may, you are its enemy."

This speaks for itself. No one, however "prone to wander," will controvert it. It is a good thing to think right—much better to act it. Let your light shine, is a divine command—to be neutral

—to be a cypher, you become a creature abhorred alike by men and angels.

"Contention is not disgraceful. Indeed, where questions of morals are concerned, it is disgraceful not to contend. He is no friend of truth who will not contend for it with all the powers God has given him, at all times and on all occasions. "He is in a fuss" is not always a reflection upon a man's character. It depends upon which side he takes in the fuss as to whether it is disgraceful or honorable. If one love the truth, and is willing to contend for it, he will often be in a fuss. It is only men of little power and little earnestness who go through the world on a smooth track. "Disgraceful to be in a fuss," said a celebrated divine of Georgia, "No, sir; the purer the man, the bigger the fuss, when truth and principle is involved."

This has the ring of the true metal—the noble, manly feeling of an honorable heart. "Afraid to be in a fuss" will do for moral cowards—but not for men who would do honor to a noble manhood.

"No institution can succeed unless it be conducted upon principles of right and justice. When these are disregarded, or set aside to subserve some purpose suggested by the passions of the hour, the enterprise must eventually fail. The man must have self-respect, and a consciousness of rectitude, if he would labor with zeal and earnestness for the success of an enterprise, and if, in the means used to procure, or in the very act of accepting a position, he violates these, his efforts will be feeble, and his failure inevitable."

Our friend, in the above, first repeats unerring truisms. Hence his conclusions are logical and true. No man can, or should, hope to succeed at the expense of those common principles underlying human action—such as common honesty and integrity of purpose. Sooner or later all hopes built upon any foundation other than rectitude and principle will bring the poor, deluded victim to grief. No position in life is worth the sacrifice of an ioto of principle or honor to secure; and he who will yield either, will find, in the end, that he has bartered integrity for no purpose but to post himself in the gaze of all men as one who is unworthy to be trusted. Death is, to the true and good man, better than disgrace.

Dr. C. R. Galloway, of Canton, Miss., says: "Gentlemen, you can count upon my influence, and the ability I may possess, be it more or less, to make your Journal a success. I hope to see it so firmly established, that no adverse minds will shake it in its onward and upward march in disseminating medical knowledge. It does seem to me that it is the very thing for the travel-worn and foot-sore doctor. The articles are short, pointed and practical."

Dr. W. F. Barr, of Abingdon, Va., says: "I am pleased with your article on "Professional Troubles," &c. I do wish that physicians would act towards each other in that conformity to the Code of Ethics—which means no more nor less than to act upon the "Golden Rule" of the New Testament, viz: "Whatsoever ye would that men should do to you, do ye even so to them." Upon this precept, given by Christ himself, the *whole* Code of *Medical* Ethics is founded. No one ought to object to it!"

Our brother is right. The difficulty is not so much that the moral power and value of the ethics is underrated by our erring brothers, but that principle—backbone, if you please—is made to yield to local surroundings and circumstances. It is too often the case that a weak man, but one of "good intentions"—a mental deficiency which, a good man has said, has "paved the way to h—l"—is made to cloak and cover up the evil deeds of his friend, and to enter the arena as a gladiator in his defence. The motto of such men is: "The law is supreme, and must be obeyed to the utmost extremity"—against my enemies, but against my friends, never! In fact, there are two roads—the one based upon law, and enforced, and the other based upon "my ring," to exempt its members from the penalties of the first. Some men hope to get to heaven on the latter, by "harping loudly" on the former.

Dr. G. F. Taylor, of Ala.—"I like the Companion well, and I think it will not be long before it will become the organ for the South."

Dr. J. B. Manson, of Ga.—"Please send me the Georgia Medical Companion. It is the best thing I ever saw—just suited to the necessities of the country practitioner."

Dr. L. B. Bouchelle, of Ga.—"I hope soon to secure several subscribers to the Companion. I think it the *very thing*—the 'one thing needful' for the working physician."

Dr. A. B. Pierce, of N. C.—"The Companion meets my wants more fully than any Journal I have ever taken."

Dr. R. C. Word, of Ga.—The last copy of the Companion was very godd. In this remark I mean no disparagement to previous ones. I think it the *most practical* Journal in the South, if not in the United States.

"The testamonials of many of the most thoroughly scientific physicians of the country, certainly attest no slight degree of merit in your journal, and the specimen copy which I have received induces me to endorse, without reservation, all that they have said in its favor. It is really a most creditable publication in every respect —in its selections, its editorials, and in its typographical excution. Mr. Toon deserves the thanks of the profession for placing in their reach a journal of such superior merit, and we trust that he will receive not their thanks alone, but will be handsomly rewarded for his exertions and trouble. Many medical journals in this country have died for lack of support. Let us trust that the one of which Mr. Toon is the publisher, will meet a different fate, as it assuredly will if it finds patronage at all commensurate with its merits. Let us, of the profession, rally to its support, make it our organ, and determine that it shall not fail."

We thank our friend for his kind words. The profession are supporting and sustaining The Companion. Our subscription list is being increased daily, and this gives us assurance to believe that its principles and devotion to *practical* medicine will make it the journal of the land. Come up friends! Roll up the largest subscription list in the country, that it may do good and circulate in every nook and corner of our land.

Dr. J. D. Tucker, of Jackson, Tenn., says: "I must say that The Companion has surpassed my anticipations. I feel sanguine that it will soon become the *most worthy and popular medical journal in the U. S., of its size.* I know several of the associate editors; they are workers of the right spirit; men who have moral worth as well as mental ability. Men who will stand upon conservative grounds in the medical profession, and will, without fear of contradiction, stand square up, in support of the *Code of Medical Ethics.* Such men are *the* men to write for a medical journal. Men who will *boldly* advocate, and endeavor to *disseminate* the grand principles of the *moral law* as a safe guide for the medical practitioner; ever *urging* that "*honesty is the best policy*" to insure a *proper* and a *worthy* success.

Every number of THE COMPANION pleases me the more. I have culled several prescriptions from its pages, that are well worth *two dollars each.* I am taking three other leading journals and prize them highly, but I would not be without THE COMPANION for any of them, as it is so convenient, and well adapted to the busy practitioner. If it maintains its present merit, I shall continue my subscription as long as I practice physic; for I shall ever be a student.

I have been, and am now, using my influence to add new subscribers to your list for the ensuing year. I trust every subscriber will do likewise. Several physicians, in the scope of my acquaintance, say they intend to send on soon. I tell every young doctor that it is especially useful and well adapted to his case, because it adheres so closely to a *moral rectitude;* and *that is the first important* lesson for him to learn. It is the *beginning* and the *end*, of the *great doctor; for to be "truly great, he must be truly good.*" "So mote it be."

DR. J. M. TAYLOR, of Corinth, Miss., says: "I desire to subscribe for your MEDICAL COMPANION. I feel that it is the duty of every Southern physician to sustain every effort looking to the establishment, and support of a journal which shall supply the wants of the profession—not on sectional or political grounds but upon the far nobler principle of self-respect, and justice to our own intelligence and enterprise. The history of similar enterprises in the South does not offer very flattering encouragement. But the bad success of former efforts does not prove the impossibility of the undertaking, but rather teaches what is required to ensure success. Your location is fortunate. I believe Atlanta possesses geographical advantages over any other point in the South, and with sufficient talent and energy your journal can be made to equal any in America. I trust you will not limit your exertions to the creation of a *Georgia Medical Journal*, but let it be an exponent of legitimate medicine *inter nos et ubique gentium.* Allow me to say, that the strongest element of support will result from medical organization. I hope it will be a prominent and leading idea in your journal to foster and encourage organization. Nothing but organized effort can ever raise us, as a profession, from the whirlpool of quackery, which is swallowing us up. Make your journal the "organ" of every medical society in the South, labor for and with them, build them up and the result will be all you wish. * * * * * * * * *

My high appreciation of the important work you have undertaken, and my sincere desire for its success, is my only apology for this communication."

We say to our esteemed correspondent, that we do not intend, as he will see from our large list of Associate and Corresponding Editors, to "limit our exertions to the creation of a Georgia Medical Journal," but that we will strive to make it, as he well suggests, "an exponent of legitimate medicine, *inter nos et ubique gentium.*" THE COMPANION is designed to meet fully the professional wants of our brethren as an out-spoken medium for them in every part of the country.

DR. J. N. BOYD, of Texas, in writing to our publisher, says: "I have been a subscriber to THE GEORGIA MEDICAL COMPANION from the first, and I do not seek to flatter when I say that I would not be without it for ten times its cost. It is just the kind of journal I need. Brief, practical, available information is what the busy practitioners of the South need, and this they will always find in THE COMPANION. Yesterday I received a package of circulars from you, and to-day I have sent them to the most worthy physicians in this and the two adjoining counties. I live twenty miles from my post-office, and do not get my mail regularly, or I suppose I would have received the package earlier. Before this, I had induced Drs. E. P. Becton, S. G. Bittick, and H. B. Lain to promise to subscribe for THE COMPANION, which I suppose they have done. My own two dollars, for 1872, I will send up in a few days, so soon as I see whether any more of the profession in this country will subscribe.

I shall have to go to Sulphur Springs, which is twenty miles from my residence, before I can get currency enough. The tax gatherer has pretty thoroughly "cleaned up" the "stamps" in this vicinity.

Rest assured I will do all I can to extend the circulation of your peerless monthly."

